# Sporadic Parathyroid Carcinoma Treated With Lenvatinib, Exhibiting a Novel Somatic *MEN1* Mutation

**DOI:** 10.1210/jcemcr/luae121

**Published:** 2024-07-24

**Authors:** Yu Ito, Toshinori Imaizumi, Hisashi Daido, Takehiro Kato, Daisuke Yabe

**Affiliations:** Department of Diabetes and Endocrinology, Gifu Prefectural General Medical Center, Gifu 500-8717, Japan; Department of Diabetes, Endocrinology and Metabolism/Department of Rheumatology and Clinical Immunology, Gifu University Graduate School of Medicine, Gifu 501-1194, Japan; Department of Diabetes and Endocrinology, Gifu Prefectural General Medical Center, Gifu 500-8717, Japan; Department of Diabetes, Endocrinology and Metabolism/Department of Rheumatology and Clinical Immunology, Gifu University Graduate School of Medicine, Gifu 501-1194, Japan; Department of Diabetes, Endocrinology and Metabolism/Department of Rheumatology and Clinical Immunology, Gifu University Graduate School of Medicine, Gifu 501-1194, Japan; Center for One Medicine Innovative Translational Research, Gifu University, Gifu 501-1194, Japan; Departments of Diabetes, Endocrinology and Nutrition, Kyoto University Graduate School of Medicine, Kyoto 606-8507, Japan

**Keywords:** parathyroid carcinoma, *MEN1*, VEGF receptor, multi-target tyrosine kinase inhibitors

## Abstract

Parathyroid carcinoma (PC) is extremely rare and is primarily treated surgically. Chemotherapy is an option for advanced stages, but no standard regimen exists. Emerging research suggests the efficacy of multitarget tyrosine kinase inhibitors (MTKIs) for PC, targeting vascular endothelial growth factor receptor (VEGFR) and platelet-derived growth factor receptor (PDGFR). A 61-year-old Japanese woman presented with a neck mass, diagnosed as PC with pleural and lumbar metastases. After parathyroidectomy and radiation for lumbar metastasis, immunohistochemistry showed VEGFR overexpression, leading to targeted therapy with MTKIs. Despite no actionable mutations on cancer genomic panel test, a novel *MEN1* somatic mutation (NM_130801: exon2: c.332delG: p.G111fs*8) was identified, which may affect VEGFR2 expression and tumor epigenetics. Although severe hand-foot syndrome necessitated dose reductions and treatment interruptions, sorafenib treatment managed hypercalcemia with evocalcet and denosumab. Lenvatinib, as second-line therapy, was effective against pleural metastases but caused thrombocytopenia and hematuria, leading to discontinuation and uncontrolled recurrence and metastasis progression. Our case highlights the need for further research on genomic profiling, molecular targets, and therapy response in PC.

## Introduction

Parathyroid carcinoma (PC) is exceedingly rare, with an annual incidence of 3.5 to 5.7 per 10 million people (1). Most cases are sporadic, with somatic mutations in genes including *CDC73* and *MEN1* (1). Currently, surgery is the only established effective treatment (2). Chemotherapy is considered for advanced cases where surgery is unachievable (2). However, owing to its rarity, no standard regimens exist (2). Recently, gene panel testing and immunohistochemical staining have guided therapy selection (2, 3). Some reports suggest the effectiveness of multitarget tyrosine kinase inhibitors (MTKIs), though resistance and side effects often necessitate medication changes or dose adjustments (2-4). Compiling case reports on treatment targets, genetic mutations, receptors, treatment responses, and adverse effects in sporadic PC is essential for developing effective therapies. Herein, we report a case of sporadic PC with a novel *MEN1* somatic mutation and vascular endothelial growth factor receptor (VEGFR) overexpression, treated with MTKIs.

## Case Presentation

A 61-year-old woman was referred to a local doctor after a left neck mass was detected during a health examination. She had a history of left kidney stones but no prior serum calcium testing. This mass was firm, elastic, and nontender. Ultrasonography revealed a hypoechoic nodule in the left neck, and blood tests showed hypercalcemia. She was referred to our hospital for further examination. Apart from the neck tumor, she was asymptomatic.

## Diagnostic Assessment

**Figure 1.**
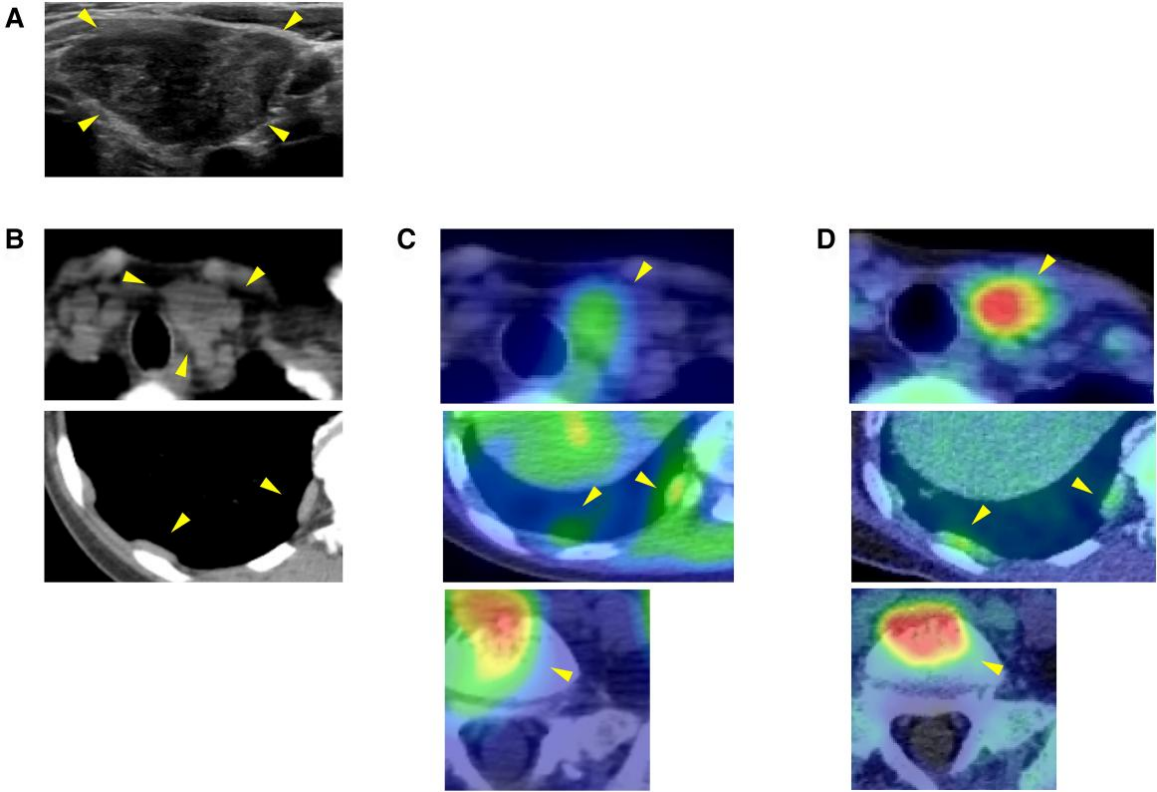
Neck ultrasonography identified a well-defined, heterogeneous lesion measuring 26 × 24 × 16 mm in the left inferior parathyroid gland (A). Chest computed tomography showed multiple pleural lesions (B). ^99m^Tc-MIBI scintigraphy (C) and FDG-PET (D) showed significant uptake in the parathyroid tumor, the area of pleural thickening, and fifth lumbar vertebra, suggesting left inferior parathyroid carcinoma with pleural and lumber metastasis. Abbreviations: FDG-PET: fluorodeoxyglucose-positron emission tomography; MIBI: methoxy-isobutyl-isonitrile; Tc: technetium.

**Figure 2.**
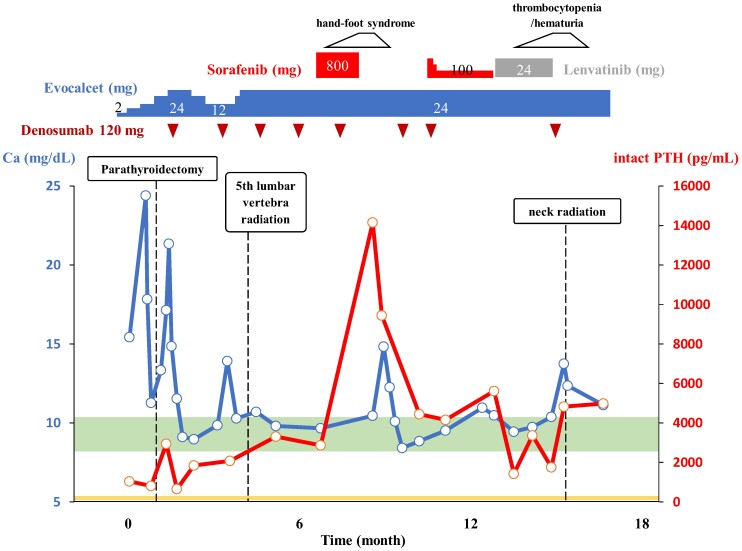
Summary of the clinical course of the PTH and serum calcium levels from the diagnosis of parathyroid carcinoma. The reference ranges for serum calcium (8.8-10.1 mg/dL [2.19-2.52 mmol/L]) and intact PTH (10-65 pg/mL [1.06-6.89 pmol/L]) are indicated by green and yellow bands, respectively. Treatment initiated with evocalcet in the outpatient setting failed to adequately correct the hypercalcemia, necessitating hospitalization. Left parathyroidectomy and left thyroid lobectomy were undertaken. Thereafter, a regimen of evocalcet, denosumab, and radiation (3 Gy/20 Fr) to the fifth lumbar vertebra was subsequently implemented. The patient then received sorafenib, but due to the onset of hand-foot syndrome, a dose reduction of sorafenib was required, and with disease progression, lenvatinib was introduced. However, lenvatinib was discontinued owing to thrombocytopenia and hematuria, following which there was a local recurrence of the parathyroid carcinoma with skin infiltration. Despite neck radiation, the treatment response was limited, and the patient died at the age of 62.

**Figure 3.**
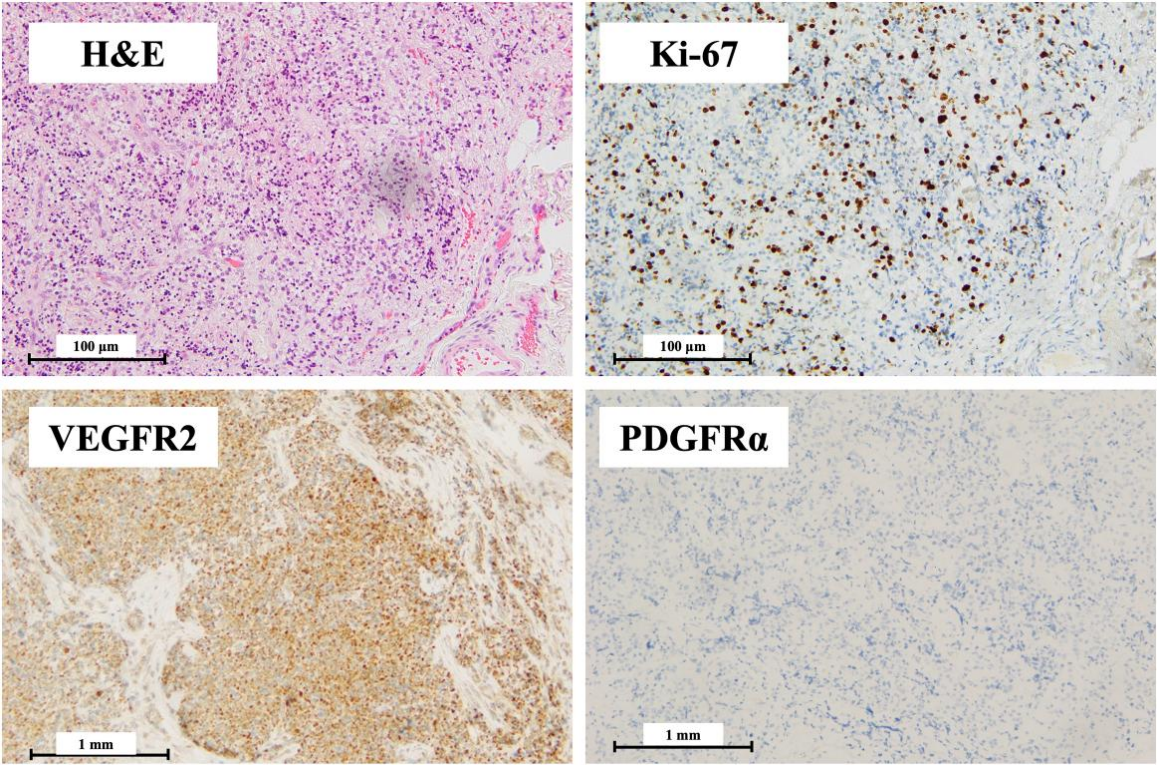
Pathological findings of the surgical excision specimen of parathyroid carcinoma. Immunostaining for VEGFR2 (monoclonal; Santa Cruz Biotechnology, Dallas, USA) and PDGFRα (monoclonal; Abcam, Cambridge, UK), which can be highly expressed in parathyroid carcinoma, was performed to investigate therapeutic targets. The results suggested that VEGFR2 was expressed, and we selected sorafenib as therapy targeting VEGFR2. Abbreviations: PDGFRα: platelet-derived growth factor alpha receptor; VEGFR: vascular endothelial growth factor.

Laboratory results showed serum corrected calcium 15.42 mg/dL (3.86 mmol/L; reference: 8.8-10.1 mg/dL, 2.20-2.55 mmol/L), phosphate 2.3 mg/dL (0.74 mmol/L; reference: 2.7-4.6 mg/dL, 0.87-1.49 mmol/L), intact PTH 1037 pg/mL (109.9 pmol/L; reference: 10-65 pg/mL, 1.06-6.89 pmol/L), and fractional excretion of calcium 5.21%, suggesting primary hyperparathyroidism (Table 1). Ultrasonography identified a well-defined, heterogeneous 26 × 24 × 16 mm tumor in the left inferior parathyroid gland (Fig. 1A). ^99m^Technetium-methoxy-isobutyl-isonitrile scintigraphy (^99m^Tc-MIBI) and fluorodeoxyglucose-positron emission tomography (FDG-PET) showed significant uptake in the parathyroid tumor and pleural thickening area, suggesting PC with pleural metastasis (Fig. 1B-1D). The electrocardiogram showed a slightly shortened but normal QT interval (QTc 361 ms) with no other abnormalities. After starting evocalcet, the patient experienced vomiting and worsened hypercalcemia (serum corrected calcium 24.41 mg/dL, 6.10 mmol/L) but remained alert. Despite aggressive hydration, medications including a single intravenous dose of 4 mg zoledronic acid, and hemodialysis, normalizing serum calcium remained challenging (Fig. 2). Subsequently, left parathyroidectomy and left thyroid lobectomy were performed. Pathological examination of the resected parathyroid tumor revealed eosinophilic tumor cells with neural and vascular invasion, and a high Ki-67 positivity rate of 20% to 33%, indicative of PC (Fig. 3). Parafibromin staining was not performed. Biopsy and surgery for the pleural lesions were risky due to dissemination concerns. FDG-PET and (^99m^Tc-MIBI) showed accumulation in the fifth lumbar vertebra, prompting a bone biopsy that confirmed metastasis (Fig. 1C and 1D). Treatment with evocalcet, denosumab, and radiation (3 Gy/20 Fr) to the lumbar vertebra controlled hypercalcemia and reduced MIBI accumulation (Fig. 3). A comprehensive cancer genomic profile (CGP) test (FoundationOne® CDx; Foundation Medicine, Inc., Cambridge, MA, USA) on the resected parathyroid tumor revealed a novel *MEN1* mutation (NM_130801: exon2: c.332delG: p.G111fs*8), and the lack of this mutation in peripheral blood sequencing with multiplex ligation–dependent probe amplification suggested its somatic nature. No *CDC73* mutation was found. Microsatellite stability and low tumor mutation burden ruled out efficacy from immune checkpoint inhibitors, and no other actionable mutations were identified. Immunostaining revealed VEGFR2 overexpression (Fig. 3).

**Table 1.** Biochemistry, urinalysis, and basal levels of various hormones

	Value	Reference range
Biochemistry		
Ca	15.42 mg/dL (3.85 mmol/L)	8.8-10.1 mg/dL (2.19-2.52 mmol/L)
P	2.3 mg/dL (0.74 mmol/L)	2.7-4.6 mg/dL (0.87-1.48 mmol/L)
Mg	1.3 mg/dL (0.54 mmol/L)	1.8-3.6 mg/dL (0.74-1.48 mmol/L)
BUN	21 mg/dL (7.497 mmol/L)	8-20 mg/dL (2.86-7.14 mmol/L)
Cre	1.08 mg/dL (95.47 µmol/L)	0.65-1.07 mg/dL (57.5-94.6 µmol/L)
Urinalysis		
Ca	0.74 mg/gCre	
FECa	5.21%	2-4%
%TRP	59.3%	80-92%
Various hormones		
Intact PTH	1037 pg/mL (109.9 pmol/L)	10-65 pg/mL (1.06-6.89 pmol/L)
PTHrP	<1.1 µg/dL (<408.5 nmol/L)	<1.1 µg/dL (<408.5 nmol/L)
1,25(OH)_2_VitD	83 pg/mL (207.17 pmol/L)	20.0-60.0 pg/mL (49.92-149.76 pmol/L)
TSH	1.15 µIU/mL (1.445 mU/L)	0.541-4.261 µIU/mL (0.541-4.261 mU/L)
Free T4	0.74 ng/dL (9.51 pmol/L)	0.76-1.65 ng/dL (9.77-21.24 pmol/L)

Values in parentheses are Système International.

Abbreviations: BUN, blood urea nitrogen; Cre, creatinine; EFCa, fractional excretion of calcium; %TRP, %tubular reabsorption of phosphate; PTH, parathyroid hormone; TSH, thyroid-stimulating hormone; T4, thyroxine.

## Treatment

**Figure 4.**
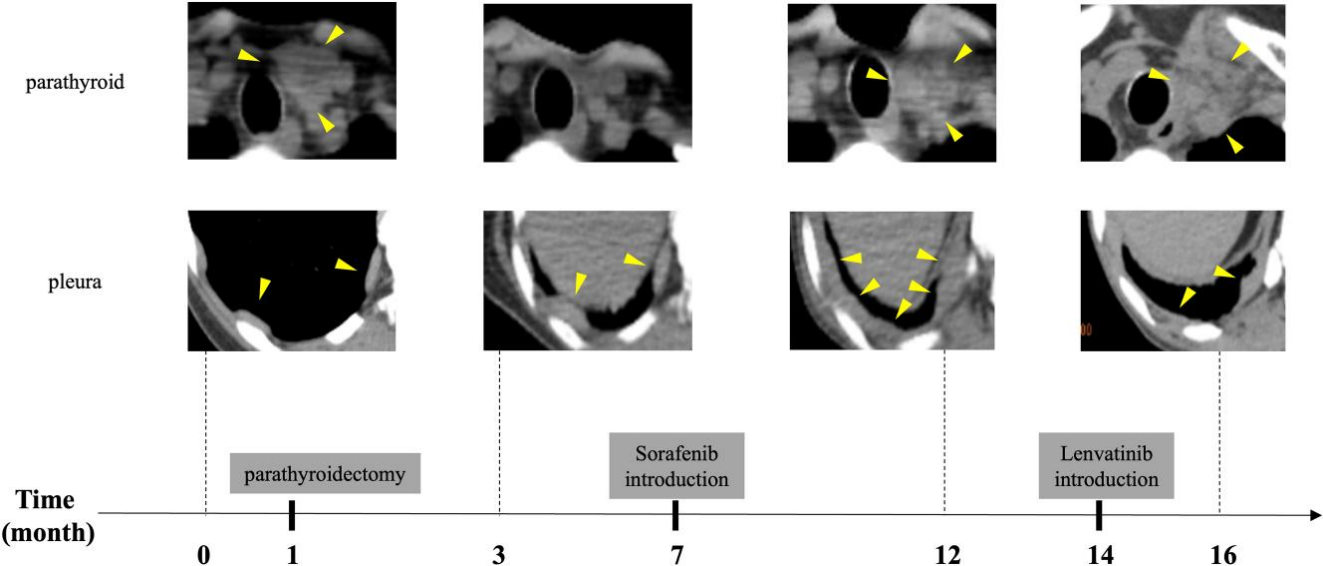
Time course of computed tomography findings of primary lesion and pleural metastasis. The primary lesion did not recur for a certain period of time after parathyroidectomy, but a local recurrence was noted around 12 months and showed a tendency to increase. On the other hand, the pleural lesions were gradually increasing, but after the introduction of lenvatinib, some areas showed a tendency to shrink.

We selected sorafenib, approved by our institution's ethics committee for off-label use in Japan, and started therapy at 400 mg twice daily. Although a dose reduction was required due to hand-foot syndrome, primary hyperparathyroidism was generally controlled with evocalcet 24 mg per day and denosumab 120 mg per month. (Fig. 2). After that, the disease progressed, and we introduced lenvatinib as second-line therapy, which possesses a more potent VEGFR inhibitory effect (Figs. 2 and 4) and is also approved by our institution's ethics committee as an off-label use.

## Outcome and Follow-up

Lenvatinib generally controlled hypercalcemia and reduced pleural metastases (Fig. 4). Subsequently, adverse events including thrombocytopenia and hematuria necessitated discontinuation, leading to local recurrence with skin infiltration. Neck radiation was administered but had limited effect. She died at age 62, with the exact cause of death undetermined due to lack of an autopsy.

## Discussion

We report on a case of sporadic PC treated with lenvatinib, exhibiting a novel somatic *MEN1* mutation. Despite presenting with relatively severe hypercalcemia, the patient surprisingly lacked typical symptoms at initial presentation. Given her history of kidney stones, she may have had prolonged hypercalcemia. The lack of symptoms could have been due to metabolic adaptation occurring in cases of chronic hypercalcemia (5).

Among treatments for PC, only surgery has established efficacy. The principle is to perform surgery on both the primary and metastatic lesions wherever possible, aiming to correct primary hyperparathyroidism and improve prognosis (2). In our case, while the parathyroid tumor was excised, complete resection of pleural and lumbar spine metastases was challenging. Therefore, radiation was administered to the lumbar spine, considering the risk of adverse events like radiation pneumonitis, despite PC's typical resistance to radiation (6). We also initiated targeted therapy to manage disease progression. Although no standard targeted therapy regimen exists, recent reports suggest immune checkpoint inhibitors and MTKIs may be effective based on CGP tests and immunohistochemistry (3).

In our case, CGP test identified no actionable mutations. A somatic mutation was found in the *MEN1* gene, which consists of 10 exons and encodes the protein menin, comprising 610 amino acids (7). Menin is primarily found in the nucleus and interacts with replication protein A, mammalian Sin3a, and mixed-lineage leukemia 2, which are related to DNA repair, cell cycle regulation, and chromatin remodeling, respectively (8). *MEN1* is a tumor suppressor gene associated with multiple endocrine neoplasia type 1 and many sporadic endocrine tumors, such as parathyroid tumors, pancreatic insulinomas, and pituitary adenomas, suggesting somatic *MEN1* mutations play a role in nonhereditary endocrine tumors (9-12). Up to approximately 35% of parathyroid adenomas have somatic *MEN1* mutations (13). In PC, there are 5.3 genetic alterations per case, including *CDC73* (38%) and *MEN1* (31%) (14). Furthermore, PC can develop in multiple endocrine neoplasia type 1 due to germline and somatic *MEN1* gene inactivation (15). The *MEN1* c.332del (p.Gly111fs) mutation, previously documented as a germline mutation in familial multiple endocrine neoplasia type 1, is identified as a novel somatic mutation in sporadic PC in our case (16). Although candidates like leflunomide, an inhibitor of dihydroorotate dehydrogenase, have been suggested as potential treatments for *MEN1* mutation-positive tumors (17), no established chemotherapeutic regimen has proven effective for sporadic PC harboring *MEN1* mutations.

Next, we performed immunohistochemistry for VEGFR and PDGFR, reported to be highly expressed in PC (18). Overexpression of VEGFR led us to introduce sorafenib, an MTKI, targeting this receptor. MTKIs inhibit multiple kinases involved in angiogenesis and cell proliferation including VEGFR and PDGFR (19). MTKIs are often used empirically for PC due to their tendency to overexpress these receptors (3). There are reports of sorafenib being used after confirming VEGFR2 expression and lenvatinib successfully controlling the disease as a second-line therapy after discontinuing sorafenib (3, 20). Meanwhile, resistance to and side effects of MTKIs are common problems in cancer treatment (4). The drug resistance mechanisms of sorafenib and lenvatinib includes epithelial-mesenchymal transition, DNA damage, ferroptosis, RNA modification, cytokine overexpression, translational modification, and self-target signal pathway (21, 22). While using MTKIs like sorafenib or lenvatinib, it is important to monitor for adverse events and adjust doses or discontinue the drug as needed. In addition, Japanese patients often experience more side effects from lenvatinib than Western patients, leading to dose reductions or discontinuation (23). In our case, sorafenib was selected as the first-line therapy, but continuation was difficult due to severe hand-foot syndrome, leading to lenvatinib as the second-line agent. This is a rare case of PC treated with lenvatinib, exhibiting a *MEN1* somatic mutation. Limited research exists on the association between *MEN1* mutations in PC and MTKI targets including VEGFR2. In cholangiocarcinoma cell lines, *MEN1* siRNA increases mRNA expression of angiogenesis-related genes including VEGFR2 (24). *MLL1*, which binds to menin, is a histone methyltransferase associated with epigenetic modifications and H3K4 trimethylation, related to cancer immunity regulation and targeted therapy resistance (25-27). Further investigation is needed on the relationship between somatic mutations in PC genes, including *MEN1*, and MTKI target molecules.

In our case, both MTKIs effectively controlled high calcium and PTH levels in combination with evocalcet and denosumab. A retrospective analysis of advanced thyroid cancer patients also showed that MTKIs reduce calcium levels through both PTH-dependent and PTH-independent mechanisms, suggesting their efficacy in correcting hypercalcemia in PC (28). Although local recurrence was not adequately controlled and lenvatinib treatment was challenging due to side effects, lenvatinib reduced pleural metastasis. Future research should explore intratumor heterogeneity in PC and differences in therapeutic target molecule expression, such as VEGFR2, between primary and metastatic lesions (29, 30).

In conclusion, we described a rare case of sporadic PC with a novel *MEN1* somatic mutation treated with lenvatinib. Despite challenges related to resistance and side effects, MTKIs showed some efficacy. Optimal management of advanced PC requires compiling case studies focused on the genetic and molecular landscape, especially elements suitable for targeted treatments. Analyzing outcomes, resistance patterns, and adverse effects associated with therapies, including MTKIs, is essential for guiding precise and effective treatment choices.

## Learning Points

- PC is a rare cancer, often involving sporadic cases with somatic mutations in genes including *MEN1*. Targeted therapy is an option for advanced cases, but no standard regimen exists.
- CGP and immunohistochemistry have guided targeted therapy selection, including MTKIs. These target VEGFR2 and PDGFRα, often overexpressed in PC, and have shown effectiveness but may require dose adjustments due to resistance and side effects.
- Compiling case studies on genetic mutations, receptors, treatment responses, and adverse effects is essential for developing effective targeted therapies. This guides precise treatment choices, especially for molecular targets and therapy responses in PC.

## Data Availability

Original data generated and analyzed during this study are included in this published article.
